# Starches, Sugars and Obesity

**DOI:** 10.3390/nu3030341

**Published:** 2011-03-14

**Authors:** Erik E. J. G. Aller, Itziar Abete, Arne Astrup, J. Alfredo Martinez, Marleen A. van Baak

**Affiliations:** 1 NUTRIM School for Nutrition, Toxicology and Metabolism, Maastricht University Medical Centre+, Maastricht, The Netherlands; Email: m.vanbaak@maastrichtuniversity.nl; 2 Department of Physiology and Nutrition, University of Navarra, Pamplona, Spain; Email: iabetego@unav.es (I.A.); jalfmtz@unav.es (J.A.M.); 3 Department of Human Nutrition, Faculty of Life Sciences, University of Copenhagen, Copenhagen, Denmark; Email: ast@life.ku.dk

**Keywords:** starch, sugars, obesity, metabolic syndrome, insulin resistance, lipids, hormones, energy intake, energy expenditure, satiety

## Abstract

The rising prevalence of obesity, not only in adults but also in children and adolescents, is one of the most important public health problems in developed and developing countries. As one possible way to tackle obesity, a great interest has been stimulated in understanding the relationship between different types of dietary carbohydrate and appetite regulation, body weight and body composition. The present article reviews the conclusions from recent reviews and meta-analyses on the effects of different starches and sugars on body weight management and metabolic disturbances, and provides an update of the most recent studies on this topic. From the literature reviewed in this paper, potential beneficial effects of intake of starchy foods, especially those containing slowly-digestible and resistant starches, and potential detrimental effects of high intakes of fructose become apparent. This supports the intake of whole grains, legumes and vegetables, which contain more appropriate sources of carbohydrates associated with reduced risk of cardiovascular and other chronic diseases, rather than foods rich in sugars, especially in the form of sugar-sweetened beverages.

## 1. Introduction

The rising prevalence of obesity, not only in adults but also in children and adolescents, is one of the most important public health problems in developed and developing countries [1]. Even though more and more people are becoming aware of the magnitude of the problem and the serious consequences associated to the development of obesity [2,3], it continues to grow. 

Lifestyle changes together with genetic predisposition play an important role in the obesity problem [4]. Large numbers of new products and technological advances have led to a lifestyle characterized by the high availability of energy-dense foods and a high level of physical inactivity [5]. Although, weight loss is usually not so difficult to attain, especially for people with a strong motivation, the real problem remains in the maintenance of the achieved body weight, since during this period individuals have to continue with habits learned during the intervention in an environment that promotes just the opposite. Indeed, the success rate over the long term is considered poor. Nevertheless, lifestyle change in diet and physical activity are still regarded as the primary strategy for weight loss, weight management, as well as for improving metabolic alterations [6]. Dietary recommendations have not changed much over the last forty years and the most commonly recommended macronutrient distribution is still 50–60% for carbohydrates, 30% for fat and 10–20% for proteins. Energy restriction and adherence to the energy-restricted diet appear to be more important than the macronutrient distribution of such a diet in causing weight loss [7]. However, increasing evidence shows that changes in protein, carbohydrate and lipid proportions could be a key factor to improve body weight regulation after a weight loss program [8]. Thus, macronutrient distribution, together with food properties (energy density, satiety value, taste, metabolic response elicited, *etc.*), are all nutritional factors conditioning energy balance. Therefore, they have the potential to contribute to better maintenance of body weight and better metabolic regulation.

**Figure 1:**
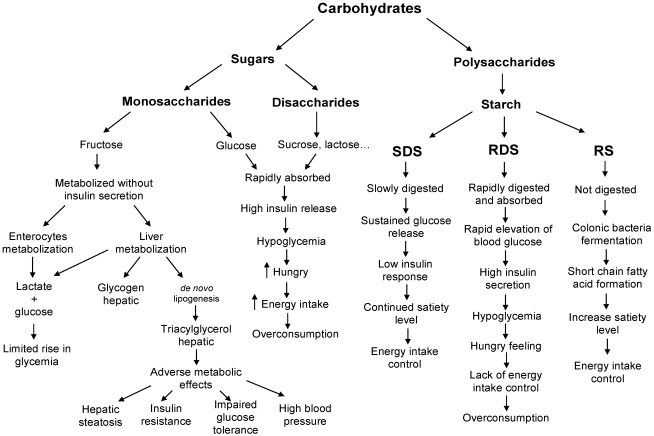
Carbohydrate classification and their main postprandial effects. SDS: slowly digestible starch; RDS: rapid digestible starch; RS: resistant starch.

Scientific evidence shows that high-fat diets have a high energy density and low satiety value, which facilitate passive overconsumption [9]. On the other hand, high-protein diets could be an alternative, since proteins have a high satiety effect that may favour energy intake control [10]. Low-fat/high carbohydrate diets also seem to be effective for body weight management [11]; however the amount and type of carbohydrates included influence the metabolic responses. Indeed, high carbohydrate and specifically high sugar consumption are often considered particularly harmful with respect to energy balance disturbances due to their specific properties related to postprandial metabolism, the balance between nutrient storage and oxidation, the effects on hunger and satiety, and hence on caloric intake and energy balance (Figure 1). 

The present article reviews the conclusions from the latest reviews and meta-analyses, if available, on the effects of different starches and sugars on parameters of body weight management and metabolic disturbances and provides an update of the most recent studies on this topic.

### 1.1. Classification of Carbohydrates

The classification of dietary carbohydrates is based on the degree of polymerization (*DP*) and type of linkage (alpha or beta). This divides carbohydrates into three main groups, sugars (DP 1–2), oligosaccharides (short-chain carbohydrates) (*DP* 3–9) and polysaccharides (*DP* > or = 10) [12]. Sugars can be grouped into monosaccharides, disaccharides and polyols or sugar-alcohols. Polysaccharides can be divided into starch and non-starch polysaccharides (Table 1).

**Table 1:** Classification of dietary carbohydrates (adapted from [13]).

Class	Subgroup	Principal components
**Sugars (mono- and disaccharides)**	Monosaccharides	Glucose, fructose, galactose
	Disaccharides	Sucrose, lactose, maltose, trehalose
**Sugar-alcohols (polyols)**		Sorbitol, mannitol, lactitol, xylitol, erythritol, isomaltitol, maltitol
**Oligosaccharides**	Maltooligosaccharides (alpha-glucans)	Maltodextrins
	Non-alpha-glucan oligosaccharides	Raffinose, stachyose, fructo- and galactooligosaccharides, polydextrose, inulin
**Polysaccharides**	Starch (alpha-glucans)	Amylose, amylopectin, modified starches
	Non-starch polysaccharides	Cellulose, hemicellulose, pectins, hydrocolloids (e.g., gums, mucilages, beta-glucans)

For the purpose of this review the term “sugars” is used for all sugars from all sources other than polyols. Specific attention will be paid to fructose, either being part of sucrose or high fructose corn syrup (HFCS), because there is (inconsistent) evidence that the consumption of fructose may be part of the cause of the obesity epidemic [14]. The term “starches” in this review addresses only the starch polysaccharides (amylose, amylopectin and modified starches).

### 1.2. Carbohydrate Digestion

Nutritional properties of carbohydrates depend on their rate and extent of digestion and absorption in the small intestine [15]. The type of monosaccharide absorbed, and the presence of other nutritional components such as fat, dietary fiber, and protein, also influences the physiological response to carbohydrates. 

Only monosaccharide species like glucose, fructose and galactose can be absorbed via active membrane transport systems. Disaccharides and polysaccharides have to be split into their monosaccharide components to be absorbed. 

The starch source, granular structure, and the degree of isolation and processing are important factors influencing starch digestion. Moreover, starches that are relatively high in amylose content tend to be more resistant to digestion than starches with higher amylopectin content. Considering this, starch can be divided into rapidly digestible starch (RDS), slowly digestible starch (SDS), and resistant starch (RS) [16]. RDS is rapidly digested and absorbed in the duodenum and proximal regions of the small intestine leading to a rapid elevation of blood glucose and usually a subsequent episode of hypoglycaemia. These rapid and large increases in blood glucose levels can further lead to cell, tissue and organ damage [17]. RS (which can also be divided into different types, see Table 2) is not digested in the upper gastrointestinal tract but is fermented by the colonic microflora, producing short chain fatty acids that provide additional energy to the body along with butyrate that is beneficial to colonic health. SDS is digested slowly throughout the small intestine to provide sustained glucose release with a low initial glycemia and subsequently a slow and prolonged release of glucose, leading to prolonged energy availability, compared to more rapidly digestible starch [15]. 

**Table 2:** Classification of naturally occurring starch (adapted from [18]).

Type of starch	Example	Probable digestion in the small intestine
**Rapidly digestible starch**	Freshly cooked starchy foods	Rapid
**Slowly digestible starch**	Most raw cereals	Slow but complete
**Resistant starch**		
**(1) Physically indigestible starch**	Partly milled grains and seeds	Resistant
**(2) Resistant starch granules **	Raw potato and banana	Resistant
**(3) Retrograded starch**	Cooled, cooked potato, bread, and cornflakes	Resistant

### 1.3. Glycemic Index and Glycemic Load

The glycemic response to ingestion of a carbohydrate depends on the amount, rate of digestion, absorption and metabolism of the ingested carbohydrate. To describe the physiological effect of a food’s carbohydrate content on postprandial blood glucose concentration, the glycemic index of foods (GI) has been introduced [19]. The glycemic index of a food quantifies the area under the glycemic response curve (AUC) of a test food, compared to the same amount (usually 50 g of available carbohydrate) of a reference food, most often glucose or white bread [19]. Generally, foods with a GI ≤ 55 are classified as low GI, whereas foods with a GI ≥ 70 are classified as high GI foods. Low-GI foods are those that elicit a low postprandial glucose response which, in turn, induces a lower rise in circulating insulin and related gastrointestinal hormones, such as incretins, gastric inhibitory polypeptide (GIP), and glucagon-like peptide-1 (GLP-1). The lower but sustained insulin secretion reduces free fatty acids levels improving cellular glucose metabolism [20]. Consequently, blood glucose levels remain closer to baseline despite continued glucose absorption from the small intestine. In contrast, high-GI foods increase insulin secretion leading to a postprandial hyperinsulinemia, which has a lipogenic effect. 

Because the glycemic response to food ingestion not only depends on the GI but also on the total amount of carbohydrates ingested, the concept of glycemic load (GL) has been introduced. Glycemic load is the product of a food’s GI and its total available carbohydrate content: GL = [GI × carbohydrate (g)]/100. Therefore, the GL provides a summary measure of the relative glycemic impact of a typical serving of the food. Foods with a GL ≤ 10 have been classified as low GL, and those with a value ≥ 20 as high GL [21].

## 2. Starches, Obesity and Factors of the Metabolic Syndrome

### 2.1. Starch Intake, Appetite, Energy Expenditure and Body Weight

It is known that an excessive intake from all macronutrients, including carbohydrates, contributes to the development of obesity. As mentioned before, starch can be divided into rapidly digestible starch (RDS), slowly digestible starch (SDS) and resistant starch (RS) (Table 2). Starch foods usually contain all three of the fractions and cannot be easily separated into pure RDS, SDS and RS foods. Many interventions have been carried out to assess the involvement of starch consumption in appetite, energy expenditure and hence, in body weight regulation. Several studies have shown that higher intakes of SDS and RS are associated with increased satiety, reduced hunger and/or reduced body weight [22]. 

#### 2.1.1. Short-Term Effects on Energy Intake and Satiety

Although the mechanisms are not totally clear, a primary mechanism by which starches are thought to regulate satiety and food intake is through their effect on blood glucose. Most nutritional intervention studies use GI to compare the effects after carbohydrate consumption. Two recent reviews concluded that consumption of high-GI foods increase hunger and decrease satiety levels in short-term human intervention studies [23,24]. Several newer studies have since addressed this issue. A randomized crossover study by Sands *et al.* [25] examined the effects of waxy maize (WM) (amylopectin, a rapidly digested starch in a slowly digested form (uncooked)) and a maltodextrin-sucrose mixture (MS) (rapidly digestible carbohydrate) or white bread (control) on postprandial insulin and glucose, whole-body energy expenditure and appetite in men and women. Twelve subjects (BMI 22.2 ± 0.7 kg/m^2^) consumed 50 g of available carbohydrate as WM, MS or white bread on separate days. Postprandial plasma glucose, insulin and appetite (hunger, fullness, and desire to eat) were measured over 4 h. The results indicated that consumption of uncooked WM, a slowly digestible starch, leads to lower postprandial glucose and insulin concentrations without an effect on appetite compared to the consumption of rapidly digested MS [25]. Another randomized crossover study by Kristensen *et al.* [26] evaluated the effect of iso-caloric meals of wholemeal wheat breads and pasta in 16 young adults, in comparison to similar refined wheat products on postprandial glycemia, appetite and *ad libitum* energy intake (EI). The meals (50 g carbohydrates) consisted of refined wheat bread (RWB), wholegrain wheat bread (WWB), refined wheat pasta (RWP) and wholegrain wheat pasta (WWP) and were served after an overnight fast. Appetite ratings and blood glucose were assessed for 180 min after which an *ad libitum* lunch meal was served and EI measured. Results showed that wholemeal breads increased satiety measures compared to their refined counterparts; however no differences were observed in the subsequent EI [26]. Likewise, another crossover study by Schroeder *et al.* [27] compared the effect of whole grain high-fiber barley, whole grain wheat and refined rice-based foods on EI and satiety. Forty-seven healthy subjects consumed a breakfast and, two hours later, a snack containing barley, wheat, or refined rice, followed 90 min later by an *ad libitum* lunch. The intake of a whole grain high-fiber barley, whole grain wheat, or refined rice breakfast and snack did not decrease energy intake at lunch [27]. A randomized crossover intervention study by Aston and coworkers [28], consisting of two consecutive 12-week periods to assess the effects of a reduced glycemic index diet on appetite, did not find differences in satiety, nor in energy intake from a midmorning snack 2 h and an *ad libitum* lunch 4 h after a high or low GI breakfast differing in GI by 12 units at the end of the intervention period in 19 subjects [28]. 

Resistant starch, a fermentable carbohydrate, has been proposed to have properties similar to dietary fiber [29] and therefore could also affect satiety and exert a beneficial role in weight regulation. Some *in vivo* studies in humans have shown that meals high in resistant starch resulted in lower glucose and insulin responses and induced greater satiety for 2–6 h [30]. Recently, a randomized double-blind, crossover study by Willis *et al.* compared the effects of four fibers and a low fiber treatment on satiety [22]. Fibers were baked in muffins and were chosen in order to represent soluble, insoluble, and RS characteristics. Twenty healthy subjects (7 men and 13 women) tested the five different muffins (identical macronutrient content) in five different visits where the satiety level was evaluated postprandially for 3 h. Results showed that subjects were less hungry after eating RS than after eating the low fiber muffin and RS stimulated less desire for food intake than the low fiber treatment. 

An acute randomized, single-blind crossover study by Bodinham *et al.* [31] aimed to determine the effects of consumption of 48 g RS on appetite, compared to an energy- and available carbohydrate-matched placebo. Twenty young healthy adult males consumed either RS or placebo divided equally between two mixed meals (breakfast and lunch) on two separate occasions. Effects on appetite were assessed using an *ad libitum* test meal 3 h after the lunch was consumed and from 24-h diet diaries for the whole 24 h of the study day. There was a significantly lower energy intake following the RS supplement compared to the placebo supplement at both the *ad libitum* test meal and over the 24 h [31]. Thus, RS intake seems to have beneficial effects on short-term satiety and food intake. 

#### 2.1.2. Effect on Energy Expenditure

The more prolonged energy availability after SDS consumption may also lead to a more extended increase in energy expenditure. However, there are few studies regarding the effects of starch consumption on energy expenditure. A crossover study by Sands *et al.* [25] examined the effects of a slowly digestible starch and rapidly digestible carbohydrate on postprandial whole-body energy expenditure and showed no differences in postprandial energy expenditure between the starches [25]. An older, highly controlled 14 day study by Raben *et al.* [32] compared the impact on energy expenditure of *ad libitum* high-sucrose *vs.* high-starch and a high fat diet in 20 normal weight or post-obese women in a randomized crossover design. The 24 h energy expenditure was significantly increased after 14 days on the sucrose diet compared with the other diets. However, this increase was explained by the increased intake of energy and fructose on this diet [32]. A review by Dulloo and co-workers of energy balance studies in animal models suggested that a tendency for sugar-fed animals to have higher metabolizable energy intakes which induced increases in metabolic rate [33]. The reviewed studies indicated that differences in energy balance between sugar and starch are small and that any increase in thermogenesis that occurred in some of the groups fed sugar-rich diets was in response to the higher energy intake rather than due to a lower efficiency of utilization of sucrose *per se* [33]. 

A study by Heijnen *et al.* described the effect of replacement of digestible starch by resistant starch on diet-induced thermogenesis (DIT) [34]. Ten healthy males consumed three test meals, consisting of diluted, artificially-sweetened fruit syrup and either 50 g raw potato starch (550 g∙RS/kg), or 50 g pregelatinized potato starch (0 g∙RS/kg) or 30 g pregelatinized potato starch plus 20 g lactulose (670 g indigestible disaccharide/kg). The meals were served in the morning after an overnight fast. Each volunteer consumed each meal twice on six separate days in random order. Metabolic rate was measured by indirect calorimetry in the fasting state and postprandially for 5 h. Results showed that the replacement of digestible starch by RS in a single meal lowered DIT, however, the ingestion of lactulose resulted in a substantial rise in DIT which was related to its fermentation [34]. A more recent crossover study by Keogh and coworkers [35] examined the effect of barley flour, high in soluble fiber and amylose, incorporated into breakfast and lunch compared with otherwise identical meals containing white wheat flour on the thermic effect of food. Fourteen healthy women consumed a test breakfast at 7:00 and a test lunch at 13:30. Energy expenditure was measured before and after the test lunch, showing no differences in the thermic effect of the foods. 

#### 2.1.3. Effect on Body Weight

Much interest has been focused on the relationship between glycemic index and body-weight loss or body weight regulation. Acute meal studies seem to point to an effect of glycaemic index on appetite and hence on body weight regulation, however, the results of longer-term studies of weight loss are not as clear. Indeed, recent conclusions from different reviews suggest that there is no evidence that an *ad libitum* diet with a low-GI causes more weight loss than a diet with a high-GI when total carbohydrate intake is not different [36]. Likewise, it is also confirmed that there is insufficient evidence that an exchange of sugar for non-sugar carbohydrates in the context of a reduced-fat *ad libitum* diet or energy-restricted diet result in greater weight reduction [37]. On the other hand, an overall systematic review suggested an overall positive effect of low glycemic or low glycemic load diets on weight loss [38]. However, no clear distinction was made between these two types of diet. 

A recent trial by Larsen *et al.* [39] studied the effect of varying protein content and GI of an *ad libitum* diet on body weight maintenance after body weight loss in 773 adults, 548 of whom completed the six month trial. Both the higher protein content and the lower GI improved completion rate and weight loss maintenance [39]. 

Animal studies observed that a diet high in RS reduced adipose tissue with no changes in body weight, suggesting a central role of short-chain fatty acids [40]. A recent study showed that an amylose-rich, high RS, *ad libitum* diet resulted in both less body weight and less body fat gain than a high amylopectin, low RS, diet [41]. In another study four groups of male C57BL/6J mice (*n* = 10 per group) were exposed to long-term (20 weeks) or short-term (6 weeks) isoenergetic and macronutrient matched diets only differing in starch type and as such GI. Mice fed the high GI diet showed a rapid-onset (from week 5) marked increase in body fat mass compared to mice in the other three starch groups [42]. In combination, the findings described above suggest that RS and SDS are attractive dietary targets for weight gain prevention and weight loss diets.

### 2.2. Starch Intake and Insulin Resistance

The starch composition of food and its rate of digestion are determinants of blood glucose and insulin levels [43]. Several studies have shown that higher intakes of slowly digested and resistant starches are associated with a reduced glycemic response and insulin resistance [22,44], while rapidly digestible starch may lead to hyperglycemic episodes, being associated with an increased risk of insulin resistance and type 2 diabetes [45].

Most of the health benefits of SDS are deduced from low-GI foods which may have a similar glycemic response as SDS. Seal *et al.* [46] showed that acute testing of SDS (corn starch) produced a slow and prolonged postprandial release of glucose and corresponding low insulin levels throughout the digestion process without a hypoglycemic effect. However, the consumption of RDS induced a rapid increase of plasma glucose and subsequent hypoglycaemia. Moreover, SDS consumption also prolonged exogenous glucose oxidation and produced a lower level of circulating non-esterified fatty acids (NEFA). 

A group of thirty-one obese subjects with elevated fasting blood glucose was randomly assigned to a two-way crossover study by Rave *et al.* [44] to evaluate the potential of a whole-grain based dietary product (WG) in comparison to a nutrient-dense meal replacement product (MR) with a similar energy but higher sugar content, during a hypo-energetic diet (4-week treatment periods with a 2-week washout period between them) on fasting blood glucose and insulin resistance. Subjects replaced at least two daily meals with WG and MR, respectively, targeting for a consumption of 200 g of either product per day. Fasting blood glucose and insulin resistance score improved during both treatment periods. However, when variables were adjusted for the amount of body weight lost, fasting serum insulin and HOMA improved more with WG than with MR [44]. Another randomized crossover study of matched diets differing only in GI and GL in 24 overweight or obese investigated the effect on insulin sensitivity. Each participant consumed both diets in random order for four weeks each, with a 4-week washout period in between. No differences in glucose metabolism factors were found [47]. Thus, although a reduction of dietary glycemic response has been proposed as a means of reducing the risk of diabetes, the impact of glycemic response on markers of health is not totally clear. Overall, Ells *et al.* [45] proposed an increase in the consumption of SDS to reduce potential risk factors for type 2 diabetes and metabolic syndrome. Indeed, several authors suggest that certain low-GI foods can lower glycemia not only in direct connection to a meal (acutely), but also at a consecutive standardized second meal, *i.e.*, lunch after a test breakfast or breakfast after a test dinner, indicating improvements in insulin sensitivity or insulin economy also within a semi-acute time frame [48,49,50,51]. In the case of benefits of breakfast into lunch, the key factor involved is likely the lente carbohydrate (capable of maintaining a low but sustained net increment in blood glucose) [49,52,53]. However, with respect to the influence of the evening meal on the response to breakfast, other properties of the low-GI foods, such as the specific amount of indigestible carbohydrates, might contribute to the improvement of glucose tolerance [48,51]. A study carried out by Nilsson *et al.* [54] to assess whether acute glycemia and glycemia after subsequent meals can be modulated by the characteristics of cereal foods, such as glycemic index and content of indigestible carbohydrates, evaluated twelve healthy subjects who had to consume test meals in random order. In series 1, test meals were consumed at breakfast and in series 2, the subjects consumed test evening meals. The authors confirmed that glucose tolerance at subsequent meals can be notably improved during the course of a whole day or overnight by choosing specific low GI, whole-grain cereal products. They also suggested that a low GI may be sufficient to achieve a second-meal effect from breakfast to lunch and a specific indigestible carbohydrate mixture appears to be required to show benefits on glucose tolerance over a longer time frame (9.5 h), most likely mediated through colonic fermentation [54]. 

In this sense, a great number of authors emphasize the positive influence on insulin sensitivity of resistant starch consumption or supplementation [55,56,57,58,59,60]. Diets rich in RS are associated with a reduced risk of diabetes. Animal studies have shown that high RS consumption improves insulin sensitivity via changes in ectopic fat storage [40,61]. However, a nutritional intervention study by Johnston, *et al.* carried out in 20 insulin resistant subjects, showed that the consumption of RS improved insulin sensitivity, but this improvement was not related to changes in body adiposity [56]. The main suggested mechanism is the colonic fermentation of indigestible carbohydrates (RS), which results in the formation of short chain fatty acids (mainly acetic, propionic, and butyric acids) [62]. These metabolites may enter the circulation, and it has been suggested that certain short chain fatty acids may exert systemic effects, including benefits on glucose metabolism [58,59]. Another study by Robertson *et al.* [59] assessed acute changes in the insoluble-fiber (resistant starch) content of the diet on postprandial glucose. Thus, ten healthy subjects consumed two identical, low-residue diets on separate occasions for 24 h (33% fat; <2 g dietary fiber). One of the diets was supplemented with 60 g RS and the following morning a fiber-free meal tolerance test was carried out. The RS consumption led to lower postprandial glucose and insulin with higher insulin sensitivity, but also enhanced carbohydrate handling in the postprandial period the following day, which was potentially due to the increased rate of colonic fermentation [58]. Likewise, a 4-week supplementation period with 30 g resistant starch/d compared with placebo resulted in higher insulin sensitivity and higher insulin sensitivity during a meal tolerance test. Moreover, despite lower insulin concentration, muscle glucose clearance during the meal tolerance test was also higher after resistant starch supplementation [59]. Brighenti, *et al.* [62] studied the “second-meal effect” of greater fermentation of high-GI and low-GI carbohydrates eaten during a previous meal. Ten healthy volunteers ate three breakfast test meals consisting of sponge cakes made with rapidly digestible, non-fermentable starch (high-GI meal), or slowly digestible, partly fermentable starch (low-GI meal). Five hours later, subjects were fed the same standard lunch. Both the high-GI and low-GI meals improved glucose tolerance at lunch, but, in the case of the high-GI meal the effect was concomitant with low non-esterified fatty acid concentrations and delayed gastric emptying. The authors concluded that fermentable carbohydrates, independent of their effect on a food´s glycemic index, have the potential to regulate postprandial responses to a second meal. 

Thus, further work needs to be done before a firm conclusion can be drawn about the optimal amount and type of dietary carbohydrate for the prevention and treatment of insulin resistance, impaired glucose tolerance and type 2 diabetes, but it is clear that SDS as well as RS are important dietary components strongly associated with health improvements and reduced metabolic risks of these prevalent diseases. 

### 2.3. Starch Intake and Lipids

Although there is strong evidence that the amount and type of fat in the diet can have strong effects on metabolism, the types of carbohydrates influencing metabolic parameters is also of great interest. In this sense, slowly digestible starch seem to be related to some benefits on lipid metabolism, while rapidly digestible starch or high glycemic index carbohydrates are associated with a higher prevalence of the metabolic syndrome [63]. 

Low glycemic index foods may alter serum lipids by prolonging absorption time, spreading the nutrient load, and modifying the endocrine and metabolite response to food [64]. It has been also suggested that low glycemic index foods or carbohydrates that are not so resistant to absorption, but rather are slowly absorbed, possess some of the features of dietary fiber in providing a substrate for colonic bacterial fermentation [64]. In the small intestine, they form lente or sustained release carbohydrates which have been related to improvements in blood lipid profiles in hyperlipidemic individuals [65]. Resistant starch may lower plasma lipid levels, especially plasma cholesterol, by delaying gastric emptying, thereby limiting hepatic lipogenesis owing to less glucose as substrate and less insulin as an activator. It may also interfere with digestive enzymes decreasing substrates availability for hepatic lipid synthesis or it may interfere with micelle formation, resulting in less cholesterol absorption. Likewise, RS may inhibit hepatic cholesterol biosynthesis due to the inhibitory effects of propionate (derived from colonic fermentation of RS) on HMG-CoA reductase activity [66].

In a randomized crossover study of matched diets differing only in GI and glycemic load (GL) by Shikany *et al.* [47], 24 overweight or obese subjects were followed for two periods of four weeks with a 4-week washout period between them. The high-GI/GL diet resulted in significant reductions in total and low-density lipoprotein cholesterol, whereas high-density lipoprotein cholesterol concentration was significantly reduced on the high-GI/GL diet compared with the low-GI/GL diet. In larger cohort studies, low glycemic index foods or low glycemic index diets have been associated with higher HDL-cholesterol levels and reduced incidence of diabetes and cardiovascular disease [67]. Likewise, in an older, metabolically controlled, study of 20 type 2 diabetic men and women fed high and low glycemic index diets for two 24-day periods by Jarvi *et al.* [68], the low glycemic index diet resulted in lower serum LDL levels in comparison with the control diet. In a study of 38 moderately hypercholesterolemic free-living men by Turley *et al.* [69], low-GI carbohydrates were increased by the use of grains, vegetable, legumes and fruit. This increased carbohydrate consumption reduced LDL and the LDL/HDL ratio with minor changes in HDL and triglycerides.

Hypocholesterolemic effects have been mainly related to RS consumption, but available information is contradictory. RS seems to decrease plasma cholesterol and triglyceride concentrations [30], but little is still known about the impact of RS on lipid metabolism. Animal studies indicate that a high RS diet leads to a lower weight of fat depots and can reduce serum total cholesterol triacylgycerol concentrations [70,71]. In humans, five weeks of RS feeding lowered fasting cholesterol and triglyceride concentrations [72]. Likewise, a study by Higgins *et al.* [30] examined the relationship between RS content of a meal and postprandial/post-absorptive fat oxidation. Twelve subjects consumed meals containing 0%, 2.7%, 5.4%, and 10.7% RS (as a percentage of total carbohydrate). Respiratory quotient was measured hourly. Breath samples were collected hourly following the meal and gluteal fat biopsies were also obtained at 0 and 24 h. Resistant starch, regardless of dose, had no effect on carbohydrate metabolism, free fatty acids or triacylglycerol concentration, nor on meal fat storage. However, data from indirect calorimetry showed that an addition of 5.4% RS to the diet significantly increased postprandial fat oxidation by 23%. Thus, the authors suggested that replacement of total dietary carbohydrate with RS increases postprandial lipid oxidation and may decrease fat accumulation in the long term [30].

The intervention study by Robertson *et al.* showed that acute changes in RS content had effects on postprandial carbohydrate concentrations, however, there was no effect on plasma triacylglycerol concentrations [58]. Eight non-diabetic subjects and four subjects with diet-controlled type 2 diabetes participated in a randomized cross-over study by Culling *et al.* [60], using a short-term intensive dietary modification to test the influence of the nature of the carbohydrates on metabolic responses. Volunteers had to follow three isoenergetic diets, each for three days: high-fat (50% energy from fat), high-starch and high-sugar (70% energy from carbohydrate each). Analysis of the variables showed that fasting triacylglycerol (TG) concentrations were greatest following the high-sugar diet, and lowest following the high-fat diet. There were no differences in TG concentration on the high-starch and high-fat diets [60]. 

Postprandial metabolism of two starches with different rates of hydrolysis *in vitro* was studied by Ells *et al.* [45]. One contained predominantly rapidly digestible starch and the other contained predominantly slowly digestible starch. Ten healthy female volunteers ate each test starch as part of a moderate fat test meal (containing 75 g test starch and 21 g fat) in a double-blind randomized crossover design. The metabolic response to each starch was measured after an overnight fast, in an acute 6 h study, before and after 14 days of daily consumption of 75 g test starch. Significantly more rapid and greater changes were found in glucose metabolism, as well as on non-esterified fatty acids, after consumption of the rapidly digestible starch. The 14 day adaptation period did not affect any of the glycemic or lipemic variables [45]. 

Thus, slowly absorbable and non-absorbable carbohydrates may all influence serum lipids and modify risk factors for cardiovascular disease. Resistant starch is associated with several changes in metabolism, which may confer some health benefits. However, the effect of the products of colonic fermentation and their relation to serum lipids requires further investigation. 

### 2.4. Starch Intake and Hormonal Responses

Starch is classified in RDS, SDS and RS in order to characterize its nutritional property since each fraction has important physiological consequences that differently affect metabolic hormones related to body weight regulation, satiety and energy intake. Because dietary carbohydrates vary in their rate of digestion and thus in glucose release and absorption, it is conceivable that the ability to stimulate incretin hormone secretion differs among the various types of carbohydrates. A recent review suggests a critical role of low-GI and fermentable carbohydrates for appetite regulation, since short chain fatty acids (SCFA), the products of carbohydrate fermentation, seem to activate gut hormone secretion, leading to appetite suppression [55]. 

The main incretin hormones are glucagon-like peptide-1 (GLP-1) and glucose-dependent insulinotropic polypeptide (GIP). Both play a role in the control of glucose homeostasis, and GIP is also implicated in the regulation of energy storage. Both hormones are secreted in response to ingestion of a meal. Other important hormones to be taken into account are leptin and ghrelin, which are antagonistic hormones with main roles in the regulation of food intake, energy expenditure and fat reserves [73]. Leptin is a hormone mainly produced and secreted by the adipose tissue, in proportion to the amount of fat stored, which contributes to the long-term regulation of body weight by decreasing food intake and increasing energy expenditure [74]. Leptin is also produced by the stomach in response to feeding, being also involved in the acute regulation of food intake acting as a satiating hormone [75]. Ghrelin is a hormone with antagonistic effects to those of leptin. It is known that ghrelin stimulates food intake and rises preprandially, initiating voluntary meals [76]. Recently, a study carried out in animals showed that diet composition influenced leptin and ghrelin production and secretion differentially. Thus, carbohydrate feeding resulted in lower ghrelin and higher leptin levels than fat feeding [77]. In a recent review [24] authors discuss the relationship between GI, leptin and ghrelin. Insulin and insulin-mediated glucose uptake and metabolism in adipose tissue affect blood leptin concentration and its diurnal pattern. The circulating ghrelin level is suppressed by carbohydrate-rich meals, presumably via glycemia and insulinemia. However, insulin-mediated leptin stimulation and ghrelin suppression *per se* are not consistent among studies. Thus, authors were not able to identify a clear relationship among GI, satiety-inducing leptin, and appetite-inducing ghrelin [24]. 

Nilsson *et al.* [78] studied the effect of eight cereal-based bread evening meals (50 g available starch), varying in GI and content of indigestible carbohydrates on incretin hormones after a subsequent standardized high-GI breakfast in healthy subjects (*n* = 15). The GLP-1 and glucose responses after the standardized breakfast were inversely related. Animal studies suggest that this effect may be mediated by bacterial colonic fermentation and formation of SCFA. Thus, low-GI whole grain foods appear to be capable of improving glycemic and satiety control not only acutely, but also at a meal 10 h later. 

A crossover study evaluated the effect of glucose and two starchy foods, varying in their content of rapidly and slowly available glucose, on plasma concentrations of GIP and GLP-1 in seven healthy volunteers (BMI 21.6 ± 1.1 kg/m^2^; age 23.4 years). Each volunteer was studied on three occasions at least one week apart. Test meals were: glucose, uncooked cornstarch (UCCS) and corn pasta (CP). *In vitro* characteristics were measured with the Englyst method, classifying the glucose fraction into rapidly available glucose (RAG) or slowly available glucose (SAG) to reflect the likely rate of release and absorption of glucose. CP contained more RAG than UCCS (89 and 28% of the total amount of carbohydrates, respectively). After the test meal was ingested, blood samples were taken at frequent intervals for 8 h. GIP concentrations were higher after ingestion of glucose than that after CP and UCCS ingestion. The intake of UCCS induced a sustained elevation of the incretin hormone of glucagon-like peptide-1 in the later stage (180–300 min), which can decrease gastric emptying and improve glycemic response [79], as well as prolong satiety [80]. 

An animal study showed that the inclusion of RS in the diet affected energy balance through its effect as stimulator of gut peptide YY (PYY) and GLP-1 expression [81]. More recently, a crossover study by Tarini and Wolever [57] evaluated the effect of fermentable fiber on gut hormone responses in healthy subjects. Thus, 12 subjects were studied for 6 h after consuming 400 mL drinks, containing 80 g high-fructose corn syrup (80HFCS), 56 g HFCS (56HFCS) or 56 g HFCS plus 24 g of fermentable fiber (inulin). Four hours after the test drink a standard lunch was served. Inulin significantly increased plasma glucagon-like peptide-1 concentrations at 30 min, and reduced ghrelin at 4.5 h and 6 h. These results support the hypothesis that dietary fermentable fiber increases the production of colonic short-chain fatty acids, which may reduce postprandial free fatty acid concentration and favorably affects the release of gut hormones that regulate food intake [57]. 

All these properties make RS and SDS attractive dietary targets for the development of weight maintenance diets and diets for the prevention and treatment of metabolic syndrome and cardiovascular risk factors.

## 3. Sugars, Obesity and Factors of the Metabolic Syndrome

The three macronutrients have different effects on satiety, with protein being the most and fat the least satiating. This hierarchy of macronutrients is also present in their thermic effect, where protein elicits the highest and fat the lowest thermic response after isocaloric ingestion [82]. Also, a diet combining a high protein and carbohydrate content results in a greater 24 h energy expenditure compared to a diet high in fat [83]. The macronutrient composition of the diet also affects the risk for cardiovascular disease. Thus, when in an *ad libitum* diet dietary macronutrient composition is varied, this can result in changes in body weight and cardiometabolic risk. As suggested before, different types of carbohydrates may also play a role. In the previous section, differences between types of starches have been discussed. Here we will discuss whether variations in the sugars content and type of sugars in the diet also play a role.

For a healthy diet, recommended dietary macronutrient composition according to the Institute of Medicine should be 10–35% protein, 20–35% fat and 45–65% carbohydrate [84]. Sugars, as part of the carbohydrates, are an important part of our diet. The Institute of Medicine advises an intake of sugars <25% of daily energy. Available data from national dietary surveys show a worldwide consumption of sugars between 10% and 21% of daily energy intake [85]. With respect to added sugars, the WHO recommends an added sugar intake of no more than 10% of daily energy. For Europe, the European Food Safety Authority (EFSA) notes that a number of European Union (EU) national authorities have established upper limits for population average intake or individual intake of added sugars <10% of daily energy, but others have not. It is also noted that the average intake of (added) sugars in some EU Member States exceeds 10% of daily energy, especially in children [86].

### 3.1. Intake of Sugars, Appetite, Energy Expenditure and Body Weight

Because different types of mono- and disaccharides have been shown to exert different effects on hunger and satiety, energy intake and energy expenditure, they may also exert different body weight effects. Therefore, modifying these types of sugars in the diet may sustain better weight management.

#### 3.1.1. Sugars, Appetite and Energy Intake

Given the different physiological effects of sugars (Figure 1), one could assume that they also have a different effect on appetite and satiety. Tappy and Lé [87] propose two potential mechanisms that could explain why fructose may elicit lower short-term satiation than equivalent doses of glucose or starches: (1) because of the more than five times lower glycemic index compared to glucose, fructose will elicit a lower glycemic response compared to an equivalent amount of glucose ingested; and (2) a meal containing fructose evokes less suppression of the orexigenic hormone ghrelin and less increase in the satiety hormone leptin than a meal containing an equivalent amount of glucose [87].

In 2009 Moran reviewed the results of preload studies comparing glucose, sucrose and fructose in either pure solutions or in mixed solutions/meals with respect to satiety [88]. Moran concluded that differences in food intake after different preloads are more related to the timing of ingestion relative to a test meal situation, whether the sugars are administered as pure sugars or as components of a dietary preload, and the overall volume of the preload than to intrinsic differences among the sugars. Moreover, the practical relevance of the results from preload studies with fructose intakes higher than normal is questionable [88]. Dolan *et al.* [89] reviewed long-term studies with dietary fructose intake up to 100 g/day. They conclude that there is no convincing evidence that such amount of fructose intake compared to sucrose or glucose is associated with an increase in food intake.

There are only a few studies that have examined the effects of other sugars on appetite. A study by Bowen *et al.* [90] investigated the short-term effects of four 1 MJ liquid preloads containing glucose, lactose, casein or whey, on appetite and energy intake. Acute appetite and energy intake was lower after consumption of lactose compared with glucose, which was consistent with differences in plasma ghrelin.

Artificial sweeteners are widely used in diet products because they are sweeter than natural sweeteners but lack the calories. Yang reviewed epidemiological and intervention studies and concluded that artificial sweeteners do not account for more weight loss compared to natural sweeteners. In some studies diet soda consumption was even associated with weight gain. Based on additional evidence from experimental studies, Yang suggests that the reason for these findings could be that artificial sweeteners do not activate food reward pathways in the same fashion as natural sweeteners. In artificial sweeteners there is a lack of calories after the sweet taste, which may result in compensatory overeating [91].

In conclusion, there is no consistent evidence that there is a difference in satiety and food intake after consumption of equal amounts of different sugars, either for the short- or long-term. On the other hand, artificial sweeteners increase appetite and the desire to eat compared to natural sweeteners, which makes their relevance for weight-loss purposes questionable. 

##### Dissolved *vs.* Solid Sugars: Effect on Appetite and Energy Intake

It has been hypothesized that solid carbohydrates suppress subjective appetite and short-term food intake more than a carbohydrate in dissolved form [92]. Akhavan *et al.* [93] therefore compared the effect of eating solid *vs.* dissolved foods. Test foods were 75 g of sucrose in solid form or dissolved in 300 mL water and an isocaloric 50/50 mixture of the monosaccharides glucose and fructose in liquid form. They found that the postprandial area under the curve of appetite was not different between the solid and dissolved forms of sugars nor was food intake from an *ad libitum* pizza lunch one hour later.

#### 3.1.2. Sugars and Energy Expenditure

Given the different metabolic pathways of sugars, different sugars may also have different effects on energy expenditure. Tappy *et al.* [94] compared the increment in energy expenditure (EE) after ingestion of 75 g of fructose compared to the same amount of glucose. Fructose increased EE significantly more than glucose. Schwarz and colleagues [95] found a similar difference after comparing intravenous fructose and glucose administration. Brundin and Wahren [96] confirmed these findings. Sharief and Macdonald [97] compared the effects of glucose with galactose, lactose, maltose, sucrose, a glucose-galactose mixture and a glucose-fructose mixture, on EE. Only sucrose and the glucose-fructose mixture showed a significant increase in EE compared to glucose [97]. Blaak and Saris [98] compared the thermogenic response to 75 g naturally enriched fructose, glucose, cane sugar, and digestible corn starch (all mixed with 400 mL water). The energy expenditure was higher after fructose and sucrose than after glucose and starch [98]. Thus, the more pronounced increase in EE after sucrose or glucose-fructose mixture ingestion is due to the fructose component. The EE increasing effect of fructose is probably due to the energy cost of fructose metabolism to glucose in the liver and continued gluconeogenesis [95].

With respect to the effect of sugars on EE it can be concluded that only fructose or mixtures containing fructose significantly increase EE compared to other sugars. 

#### 3.1.3. Sugars, Body Weight and Body Composition

Three reviews concerning this topic have been published recently. In the first review by van Baak and Astrup [37], the authors conclude that observational studies show fairly consistent inverse associations between the carbohydrate content and content of sugars in the diet and body weight and adiposity measures. This is supported by a limited number of randomized controlled trials (RCTs) that consistently show lower body weight when fat in the diet is replaced by carbohydrates, whether in the form of sugars or as starches. In the second review, Ruxton *et al.* [85] support these findings. The third review by Dolan *et al.* [89] focuses on dietary consumption of fructose. Here it is concluded that there is no convincing evidence from long-term studies that fructose ingestion of up to 100 g/day instead of glucose or sucrose is associated with an increase in body weight. 

A recent weight-loss intervention study, among 169 overweight/obese Scottish women, supports these conclusions. The study sample was divided into three groups. Group 1 received advice to reduce energy, total fat and sucrose for three months; Group 2 received advice to reduce energy and total fat and maintain sucrose intake at 10% energy for three months; and Group 3 acted as controls and received no dietary advice. Both Groups 1 and 2 were successful in reducing energy intakes and the percentage energy from fat in their diet. Group 1 was also successful in reducing percentage energy from sucrose at three months. These dietary changes resulted in significant reductions in body weight, percentage body fat and the waist-to-hip ratio in both groups, but there was no significant difference in weight-loss between Group 1 and 2. Reducing sucrose consumption to below 10% of total energy therefore did not lead to extra weight loss [99]. 

##### 3.1.3.1. Role of Sugar-Sweetened Beverages

Consumption of sugar-sweetened beverages (SSB’s) is often linked to an excess in caloric intake and the increasing prevalence of obesity. It is hypothesized that the calories in SSB’s have little effect on satiety and therefore easily lead to over-consumption [92]. SSB’s are defined as drinks with added sugars, excluding milk and pure fruit juices. 

In their review on sugars and body weight, van Baak and Astrup [37] concluded that a limited number of randomized controlled trials supported the positive association between BMI and SSB consumption that is found in observational studies, although not consistently [39]. A number of additional reviews on this topic has been published in the last two years [37,100,101,102,103]. In 2008, Gibson [101] published a systematic review on 23 cross-sectional, 17 prospective and four intervention studies in adults and children, as well as six reviews, using BMI, weight (gain) or adiposity as endpoints. She concludes that there is little evidence from epidemiological studies that SSB’s are more obesogenic than any other source of energy and that there is a need for more intervention studies, especially among overweight consumers of SSB’s, which use reliable measurements of diet and physical activity, and with an adequate length of follow-up [101].

A review of the literature between 1966 and 2006 on the relationship between SSB’s and weight gain by Wolff and Dansinger [102] revealed that six of 15 cross-sectional and six of 10 prospective cohort studies identified statistically significant associations between soft drink consumption and increased body weight. There were five randomized clinical trials; the two that involved adolescents indicated that efforts to reduce sugar-sweetened soft drinks slowed weight gain. In adults, three small experimental studies also suggested that consumption of sugar-sweetened soft drinks caused weight gain. None of these trials in adults however was longer than 10 weeks and they all had a rather small study population. The authors conclude that although soft-drink consumption has increased over the last decades, the evidence of SSB-related weight gain is weak. In conclusion, they call for more comprehensive intervention trials designed to evaluate the effects of soft drink consumption on body weight and cardiovascular risk factors. 

Olsen and Heitman [103] (literature up to 2007 but mostly overlapping with Wolff and Dansinger [102]), concluded that a high intake of calorically sweetened beverages can be regarded as a determinant for obesity. However, there seems to be no support that the association between intake of calorically sweetened beverages and obesity is mediated by increased energy intake, and alternative biological explanations should be explored.

Most recently, Malik *et al.* [100] concluded that SSB intake is a significant contributor to weight gain, in part because of incomplete compensation for liquid calories at subsequent meals. They also report that longer studies, with a greater numbers of participants, which do not adjust for potential mediators of effect such as energy intake, would report stronger and more consistent results. This literature suggests that the evidence of a relationship between SSB consumption and weight gain is inconclusive.

##### 3.1.3.2. Dissolved *vs.* Solid Sugars

Liquid foods may have a different effect on satiety and food intake compared with solid foods. One explanation for this difference is the absence of chewing when ingesting beverages, which may result in decreased pancreatic exocrine and endocrine responses compared with the ingestion of solid foods. Beverages are also emptied from the stomach at a higher rate than solid foods and may induce weaker signals in the gastrointestinal tract that would lead to inhibition of food intake [104].

Van Baak and Astrup most recently reviewed this topic and concluded that, from RCTs, there is no support for the hypothesis that sugars in liquids have a detrimental effect on body weight compared with solid forms of sugar. Evidence from a small number of acute studies with respect to satiety and energy intake compensation is equivocal. They call for more RCTs of sufficient size and duration in this area [37]. Since then, there has been only one RCT comparing ingestion of calories in solid form or dissolved in liquids on body weight change.

Chen *et al.* [104] compared the intake of liquid calories with solid calories on body weight change within the PREMIER trial population. PREMIER is an 18 month multicenter randomized trial designed to test the blood pressure lowering effects of two multicomponent behavioral interventions in adults with prehypertension or stage 1 hypertension. Liquid calorie intake was calculated as the sum of calories from seven beverage categories (including SSBs, diet drinks, milk, 100% juice, coffee and tea with and without sugar, and alcohol). Solid calorie intake was calculated by subtracting liquid calories from total calories. A reduction of 100 kcal/day in liquid calorie intake was associated with 0.3 kg of weight loss at six months and of 0.2 kg at 18 months. A reduction in solid calorie intake of 100 kcal/day was associated with a 0.06 kg weight loss at six months and of 0.09 kg at 18 months. Reducing liquid calorie intake resulted in more weight loss than a reduction in solid calorie intake, but only the difference at six months reached statistical significance. The comparison of the individual beverages showed that only SSB intake was significantly associated with weight change. A reduction in SSBs by one serving/day (355 mL) was associated with a weight loss of 0.5 kg at six month and of 0.7 kg at 18 months [104]. 

Although the study by Chen is about liquid *vs.* solid calories rather than sugars, it does add to the body of evidence that a reduction in liquid calories, which were mainly derived from SSB intake, has a greater effect on weight loss compared to a reduction in solid calories.

### 3.2. Sugars and Insulin Resistance

Insulin resistance and impaired glucose tolerance are common co-morbidities in the obese population. Sugars are known to have a negative influence on the incidence of type 2 diabetes. Over the last years, several large studies have been conducted to study the relationship between sugar intake and the incidence of type 2 diabetes.

Laville and Nazare [105] reviewed 21 intervention-, prospective- and cross-sectional trials between 1979 and 2007 on the relationship between sugars, insulin resistance and diabetes to determine the level of proof concerning the association of sugars consumption and diabetes [105]. They concluded that these studies failed to demonstrate an obvious relationship between the total intake of sugars and glycemic control, or risk to develop a type 2 diabetes and particularly specific evidence is missing in terms of sucrose effect on diabetes. Concerning fructose, there are still discrepancies between studies about the long-term deleterious effect on diabetes development. But its effect on lipogenesis and triglyceridemia has to be taken into account, considering the growing use of sugar, and thus fructose-containing foods [105]. 

To determine the association between surrogate markers of insulin resistance (fasting insulin, fasting glucose, homeostatic model assessment of insulin resistance (HOMA-IR), and the insulin sensitivity index (ISI_0.120_)) and SSB consumption, Yoshida *et al.* [106] used data from 2500 subjects with an average age of 54 years from the Framingham Offspring Study. 53% of the study population consumed SSB’s and did this with an average of two servings per week. After adjustment for potential confounding variables, the frequency of SSB intake was positively associated with fasting insulin. The associations between the frequency of SSB consumption and fasting plasma insulin and HOMA-IR remained statistically significant after further adjustment for dietary glycemic index, fruit intake, or vegetable intake. No significant associations were found between SSB intake and fasting glucose or ISI_0.120_. In this study, the HOMA-IR largely reflected fasting insulin concentrations. Both insulin resistance and β-cell dysfunction precede type 2 DM, and thus increased consumption of calorically sweetened beverages containing rapidly absorbable simple sugars may contribute to an increased risk of type 2 DM [106].

Teff  * et al.* [107] compared the effect of glucose and fructose, consumed in the form of sweetened beverages with isocaloric mixed nutrient meals, on circulating hormones and lipids in obese men and women. In comparison with glucose, consumption of fructose-sweetened beverages results in decreased insulin secretion, a reduced diurnal leptin profile, and increased postprandial TG concentrations in obese individuals, independent of insulin sensitivity. In addition, the effect of fructose to increase TGs was augmented in obese subjects with insulin resistance. These data suggest that overconsumption of dietary fructose may exacerbate the adverse metabolic profiles in obese individuals, particularly those with existing insulin resistance and may therefore increase the risks for developing diabetes and CVD [107].

Malik and colleagues [108] published a meta-analysis on SSB intake and risk of metabolic syndrome and type 2 diabetes. For their analysis they included 11 prospective cohort studies, eight studies with endpoint type 2 diabetes and three studies with endpoint metabolic syndrome, in total more than 310,000 individuals. They compared the most extreme categories of SSB intake, being none or one serving/month with 1–2 serving/day. According to their analysis, subjects in the highest category, had a 20% greater risk of developing the metabolic syndrome and 26% greater risk of developing type 2 diabetes, compared to subjects in the lowest category. 

From the above literature, it can be concluded that when data of prospective cohort studies are combined in a meta-analysis, an increased sugar intake, being either glucose or fructose and mostly in the form SSBs, increases the risk of developing insulin resistance and type 2 diabetes, especially in the obese. However, when single intervention, prospective and cross-sectional trials are reviewed, this relationship is often not found [105]. Also, fructose seems to have more detrimental effect on developing insulin resistance than glucose.

### 3.3. Sugars and Serum Lipids

Although the mechanism of how carbohydrates affect serum lipids is not completely understood, it has been suggested that these effects could be mediated by fructose. Fructose has been shown to increase *de novo* lipogenesis and triglyceride synthesis in the liver, and secretion of very low-density lipoproteins. Fructose also appears to decrease the peripheral clearance of lipids [109].

Dolan *et al.* [89] reviewed the effect of dietary fructose on triglyceride levels in short- and long-term feeding studies in healthy normal-weight individuals. They concluded that the majority of acute studies show a slight increase in postprandial plasma TG levels after ingestion of fructose compared to other types of carbohydrates. Schaefer and colleagues [110] support these findings. In the long-term (>1 day) however, Dolan *et al.* [89] report that there is no evidence that plasma TG are increased after ingestion of up to 133 g/day fructose in women and 136 g/day fructose in men, when it is not consumed in caloric excess. Schaefer *et al.* [110] on the contrary found significant increases in fasting TG and LDL cholesterol concentrations by fructose in 4- to 6-week studies directly comparing glucose and fructose at 20–25% of total energy intake. No metabolic differences were found between sucrose and high-fructose corn syrup (HFCS). Another review by Johnson *et al.* [109] also concluded that sugar intake appears to be associated with increased TG levels, but that the effects of sugar intake on HDL-C and LDL-C remain unclear.

Recent studies add to this evidence. Higher intakes of sugars from the candy/soda food group, from fruit-containing beverages, and from sweetened dairy foods are associated with significant increases in TG, VLDL-C and HOMA-IR in high BMI African American children [111]. Although the authors did not divide sugars into glucose or fructose, it can be assumed that their sugars are mostly fructose, because candy/soda, fruit-containing beverages and sweetened dairy foods are mostly sweetened with sucrose. A large cross-sectional study among 6113 U.S. adults revealed that increasing added sugar consumption is associated with lower HDL-C levels, higher TG levels, and higher ratios of TG to HDL-C [112]. Duffey and colleagues [113] analyzed data from the CARDIA (Coronary Artery Risk Development in Young Adults) study. They reported that a higher SSB consumption was associated with increased risk of developing high TG and high LDL-C.

These studies suggest a relationship between added sugars and increases in TG, VLDL-C and LDL-C, and decreased HDL-C levels. The effects appear to be related to the fructose in the added sugars. Since added sugars have a detrimental effect on serum lipids they are likely to increase the risk of cardiovascular disease.

### 3.4. Sugars and Blood Pressure

Elevated blood pressure is one of the components of the metabolic syndrome. Already in the 1960s, a relationship between sugar consumption and blood pressure was suggested [114]. Since then, this has been a much debated issue. Johnson [115] reviewed several possible mechanisms through which sugars, and in particular fructose, can cause hypertension. One of these mechanisms is that fructose causes an increase in uric acid concentrations that subsequently causes a reduction in the concentrations of endothelial nitric oxide, which is linked to hypertension [115].

Johnson *et al.* [109] conclude in their review that the results from human studies are inconsistent and that the chronic effects of a high intake of simple sugars on blood pressure remains uncertain.

A number of additional studies on the effect of sugars consumption have been published more recently. Bidwell and colleagues [116] studied the effects of either a glucose (100 g dextrose in 300 mL water) or isocaloric glucose-fructose (glucose:fructose; 45:55 g in 300 mL) beverage on posprandial endothelial function and blood pressure. No differences in postprandial endothelial function and blood pressure responses between the two beverages was found [116]. In a prospective cohort study among 2774 young adults, Duffey *et al.* [113] found an increased risk for hypertension with higher SSB consumption. Jalal *et al.* [117] investigated whether increased fructose intake from added sugars was associated with an increased risk for elevated blood pressure using cross-sectional data from NHANES involving 4528 adults without history of hypertension. After adjustment for confounders, increased fructose intake of ≥74 g/day was associated with higher odds of elevated BP levels (26, 30, and 77% higher risk for BP cutoffs of ≥135/85, ≥140/90, and ≥160/100 mmHg, respectively).

There is only a limited number of RCTs that investigated the effects of sugars on blood pressure. These studies do not provide sufficient evidence for a positive relationship between total intake of sugars and blood pressure. Larger well controlled RCTs should be conducted to reveal whether there is a relationship or not, and whether there is a specific role for dietary fructose consumption in this relationship.

### 3.5. Intake of Sugars and Hormonal Responses

Insulin, leptin, peptide YY, ghrelin and glucagon-like peptide-1 (GLP-1) are hormones that all have an effect on food intake regulation and/or satiety. Differences in these hormones after consumption of different sugars may contribute to their effects on body weight and metabolic risk. Most studies compared fructose (as part of HFCS or sucrose) with glucose.

Stanhope [118] reviewed the endocrine and metabolic effects of beverages sweetened with glucose, fructose or high-fructose corn syrup. They conclude that in both short-term and long-term studies fructose consumption compared to glucose consumption resulted in decreased circulating levels of insulin and leptin. Long-term high fructose consumption could therefore lead to increased caloric intake or decreased energy expenditure, and contribute to weight gain and obesity.

No studies were found that compared the effect of different sugars on PYY in humans. In rats, circulating total PYY levels were significantly increased in rats drinking glucose or sucrose (and not fructose) for 24 h compared to rats drinking water. These effects disappeared after one week. After two weeks PYY levels were significantly lower for all sugar solutions compared to water. Two weeks of fructose drinking significantly increased serum ghrelin levels compared to glucose, sucrose or water. After two weeks, serum leptin levels were significantly increased after consuming all sugar solutions compared to water. The authors propose that the increased levels of total ghrelin and decreased levels of total PYY after two weeks suggest that the rats are receiving signals to continue eating, thus promoting the hyperphagia induced by the sugar solutions [119].

Melanson  * et al.* [120] reviewed short-term studies comparing the effects of HFCS and sucrose on plasma insulin, ghrelin, leptin and GLP-1, but did not find any differences. Teff *et al.* [121] studied the effects of consuming fructose- or glucose-sweetened beverages with meals in 12 normal weight women on circulating glucose, insulin, and leptin concentrations as well as ghrelin, GLP-1, and GIP over a 24 h period. When fructose-sweetened beverages were consumed with meals, this resulted in lower circulating insulin and leptin and higher ghrelin and TG levels compared with consumption of glucose-sweetened beverages [121]. 

From the above we can conclude that the hormonal responses of fructose compared to glucose could promote the development of obesity when fructose in consumed rather that glucose.

## 4. Conclusions

There are only a few studies that have directly compared the effect of dietary intake of sugars and starches on body weight and parameters of the metabolic syndrome. A study by Raben *et al.* [32] studied the replacement of dietary fat by sucrose or starch on 14 days *ad libitum* energy intake and body weight in 20 healthy normal-weight, partly post-obese, subjects. Body weight and fat mass decreased significantly on the starch diet (by 0.7 ± 0.2 kg and 0.4 ± 0.1 kg, respectively, *P* < 0.05), whereas no changes were observed on the fat or sucrose diets. However, it is not clear whether the between-group differences were statistically significant. The starch-rich diet was also associated with lower total cholesterol, LDL, fasting and non-fasting TG, and nonfasting FVIIc than the sucrose-rich diet [122]. In the CARMEN trial [11], participants randomized to an *ad libitum* reduced fat, high starch diet lost 1.8 kg, whereas those randomized to the *ad libitum* reduced fat, high simple carbohydrates diet lost 0.9 kg (between-group difference not statistically significant). Thus, in both studies body weight loss was larger on an *ad libitum* high starch diet than on a high sugars diets and are suggestive for a more beneficial effect of starch than sugars intake on body weight. The combination of literature reviewed in this paper, suggesting potential beneficial effects of intake of starches, especially those containing slowly-digestible and resistant starches, and potential detrimental effects of high intakes of fructose, support the intake of whole grains, legumes and vegetables. These contain more appropriate sources of carbohydrates associated with reduced risk of cardiovascular and other chronic diseases, rather than foods rich in sugars, especially in the form of sugar-sweetened beverages.
